# Rat Calvarial Guided Bone Regeneration Model: Preclinical Insights into Biomaterials, Barrier Design, and Systemic Modulators

**DOI:** 10.3390/jfb16120438

**Published:** 2025-11-25

**Authors:** Akira Hasuike, Taito Watanabe, Shin Wakuda, Tomoe Suzuki, Shuto Kikuchi, Seiko Min, Yoshinori Arai, Shuichi Sato

**Affiliations:** 1 Department of Periodontology, Nihon University School of Dentistry, Tokyo 101-8310, Japan; watanabe.taito@nihon-u.ac.jp (T.W.); wakuda.shin@nihon-u.ac.jp (S.W.); deto24012@g.nihon-u.ac.jp (T.S.); desh24008@g.nihon-u.ac.jp (S.K.); satou.shuuichi@nihon-u.ac.jp (S.S.); 2 Dental Research Center, Nihon University School of Dentistry, Tokyo 101-8310, Japan; 3 Division of Applied Oral Sciences, Nihon University Graduate School of Dentistry, Tokyo 101-8310, Japan; 4 Department of Periodontics and Dental Hygiene, University of Texas Health Science Center at Houston, Houston, TX 77054, USA; seiko.s.min@uth.tmc.edu; 5 Department of Oral and Maxillofacial Radiology, Nihon University Graduate School of Dentistry, Tokyo 101-8310, Japan; arai.yoshinori@nihon-u.ac.jp

**Keywords:** guided bone regeneration, rat calvarial model, bone substitutes, barrier membranes, growth factors, systemic conditions, carbonate apatite, spatial omics

## Abstract

Guided bone regeneration (GBR) plays a key role in alveolar ridge augmentation and implant therapy, but the biological mechanisms governing its outcomes are not fully understood. Preclinical animal models provide critical insights that cannot be obtained in early human studies. Over the past 15 years, our group has developed and optimized a standardized rat calvarial GBR model using plastic caps, enabling reproducible and quantitative evaluation of bone regeneration through micro-computed tomography and histomorphometry. The present narrative review synthesizes the findings from our body of work. Our studies demonstrated that advanced substitutes such as hydroxyapatite/collagen composites and carbonate apatite provide favorable outcomes, indicating that local and systemic application of growth factors or parathyroid hormone can markedly enhance augmentation, and that barrier permeability critically modulates angiogenesis and osteogenesis. Moreover, systemic conditions such as nicotine exposure and estrogen deficiency profoundly compromise regenerative outcomes but can be partly mitigated by pharmacological interventions. Finally, regenerated bone within GBR spaces is biologically competent, although it remains less mature than native cortical bone. Together, these insights highlight the translational value of our GBR model and indicate the integration of spatial omics for the elucidation of the cellular mechanisms that will guide future regenerative strategies.

## 1. Introduction

Guided bone regeneration (GBR) is a well-established and widely utilized strategy for alveolar ridge preservation and augmentation [1,2,3]. It plays a key role in regenerative dentistry and implant therapy, for both vertical and horizontal ridge augmentation. To achieve predictable clinical outcomes, the selection of biomaterials such as barrier membranes and bone graft substitutes must be guided by sound biological evidence [4,5,6,7]. Additionally, the integration of patient-specific local and systemic risk factors into treatment planning is essential for maximizing the likelihood of long-term treatment success [8].

Although clinical application of GBR has advanced with the development of various biomaterials and surgical techniques, in many cases, its underlying biological mechanisms remain poorly understood and insufficiently validated [9]. In the absence of robust biological evidence, the implementation of these approaches in clinical practice remains ethically and scientifically challenging. Preclinical studies using standardized animal models play an essential role in addressing this issue [10]. Despite growing ethical concerns and the pursuit of alternatives to animal testing, animal experiments continue to serve as a critical bridge between basic research and clinical implementation. In regenerative medicine, including GBR, the use of animal models enables researchers to evaluate the safety, efficacy, and biological behavior of new materials and techniques under controlled conditions. Importantly, preclinical animal studies offer several advantages over human trials, such as the ability to harvest tissues for direct histological and histomorphometric analysis. These investigations establish the foundation for a stepwise translational pathway: beginning with proof-of-principle in animal models, followed by small-scale human pilot studies, eventually leading to multicenter randomized controlled trials. Ultimately, rigorous validation in preclinical models is not only scientifically necessary but ethically imperative before the clinical introduction of GBR strategies.

One of the most frequently used animal models for vertical bone augmentation is the calvarial model. The calvaria was selected because it provides a flat, thin, and easily accessible bone surface, making it well-suited for the placement of a standardized mechanical barrier to achieve vertical augmentation [11,12,13]. In GBR research, it is crucial to evaluate new bone formation within a standardized and protected space, and the calvarium offers favorable anatomical conditions for achieving this goal. Because the calvaria is a non-load-bearing site, mechanical interference during healing is minimized, and intramembranous ossification allows direct assessment of biological processes relevant to craniofacial bone augmentation.

To perform the above-described rigorous validation of GBR methods, our research group has employed a standardized plastic-cap calvarial model in rats for over 15 years. This reproducible model allows quantitative and longitudinal assessment of bone regeneration using micro-computed tomography (micro-CT) and histomorphometric analysis. The present narrative review summarizes the body of evidence derived from our rat GBR model, compares it with research evidence obtained from other calvarial models for vertical GBR, and provides a comprehensive understanding of how biomaterials, biological modulators, systemic conditions, and adverse factors influence the outcomes of bone augmentation. Drawing on our extensive body of preclinical research, we thematically synthesize findings across six domains: barrier membranes and cap design, bone substitutes, growth factors and hormones, systemic influences, adverse factors, and the osteogenic quality of regenerated bone.

## 2. Experimental Model

Our research group initially employed a rabbit calvarial model to study vertical GBR [14,15]. In our rabbit studies, we demonstrated that even an empty titanium dome placed on the calvaria can stimulate bone formation beyond the skeletal envelope, leading to de novo bone generation in areas where bone had not previously existed [15]. The newly formed bone appeared to originate from marrow perforations and the endosseous regions within the circular grooves used to secure the titanium dome. This response was likely driven by the local release of growth factors from the cortical surface, endosteal area, and surrounding marrow. Thus, our experimental GBR model effectively promoted the invasion of mesenchymal progenitor cells from the marrow compartment into the augmented space. Recently, Min et al. extended this approach by incorporating an antibody-mediated osseous regeneration (AMOR) within the same rabbit calvarial model [16]. In their study, titanium domes were filled with scaffolds functionalized with anti-BMP-2 monoclonal antibodies, with or without decortication, to enhance the capture and local activity of endogenous BMPs. Remarkably, sites treated with both decortication and AMOR exhibited the highest levels of de novo bone formation, even at locations distant from the native calvarium. These findings highlight the synergistic effects of decortication and endogenous growth factor immobilization in promoting vertical bone augmentation beyond the skeletal envelope.

Although this rabbit model provided a stable space for vertical augmentation and facilitated the evaluation of bone formation, several limitations emerged. First, the use of medium- to large-sized animals has become increasingly restricted owing to regulatory and ethical considerations. Second, while the advent of microfocus computed tomography (micro-CT; R_mCT) has enabled high-resolution, longitudinal monitoring of bone regeneration in vivo, micro-CT is, in most cases, limited to small animals and currently is not feasible in rabbits or larger species [17]. To overcome these limitations, our group established a standardized rat calvarial GBR model using a plastic cap composed of poly(methyl methacrylate) (PMMA)—an acrylic-based polymer resin. PMMA is a synthetic polymer that has attracted considerable attention in bone tissue engineering and is widely used as a bone substitute or filler, with a long history of successful application, particularly as bone cement in orthopedic and dental surgery [18,19]. The major advantages of PMMA include its biocompatibility, ease of handling, processability, dimensional stability, and low cost. In addition, it enables quantitative, longitudinal, and noninvasive evaluation of bone regeneration using micro-CT in vivo. PMMA provides a mechanically stable and chemically inert environment that maintains a defined space for bone regeneration without being resorbed or degraded. However, owing to its bioinert nature, it does not chemically bond or integrate with bone tissue, which may limit osteoconductivity and interfacial strength. In the experimental setting using animal models, mechanical loading is not applied to the calvarial site; therefore, stability is not a critical issue. Nevertheless, because PMMA cannot withstand autoclave sterilization owing to its thermal sensitivity, gas sterilization is required prior to use.

Kochi et al. validated this approach by showing that bone defects covered with a plastic cap and filled with a bone substitute supported vertical bone formation beyond the skeletal envelope [20,21]. A 20 mm midline incision was made along the sagittal suture, and two critical-size defects (5 mm in diameter) were trephined into the parietal bone on each side of the midsagittal suture, carefully avoiding damage to the dura mater and the superior sagittal sinus. The midsagittal suture was deliberately excluded from the defects to limit its influence on healing and to minimize surgical risk. In contrast to our rabbit experiments, where titanium caps served as mechanical barriers, we opted for plastic caps in the rat model to enable micro-CT imaging because titanium interferes with radiographic visualization and quantification owing to its high radiodensity. The plastic caps were fixed over the defects using light-cured composite resin, ensuring that they did not impinge upon the dura. At the experimental sites, the resin caps were filled with high-porosity hydroxyapatite (HA) granules (porosity 82.5%, pore size 600–1000 µm, weight 8 mg; Apaceram-AX, Pentax Corp., Tokyo, Japan), while control sites were left empty. Longitudinal R_mCT imaging revealed that bone formation began at the defect margins and advanced vertically into the HA scaffold, ultimately achieving significant vertical augmentation. These foundational studies validated both the biological efficacy of GBR with an HA scaffold and the technical feasibility of the rat calvarial GBR model.

**Figure 1:**
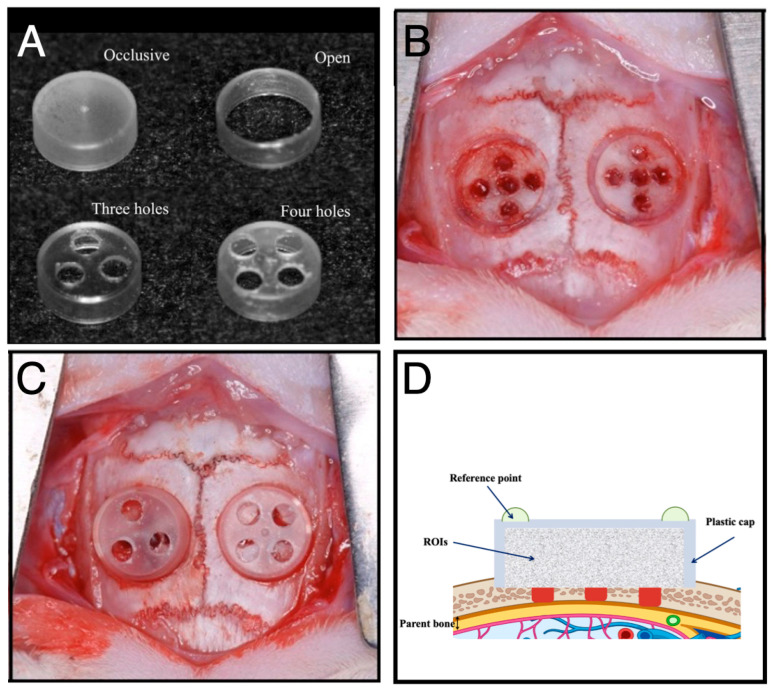
Overview of the rat calvarial GBR model. (**A**) Photograph of a plastic cap. Plastic caps with diameters ranging from 4.4 to 5.0 mm and heights ranging from 1.5 to 3.0 mm were used in our rat GBR model. (**B**) A circular groove made with a trephine bur, and five small holes drilled with a round bur, to allow marrow penetration. (**C**) Plastic caps placed on all defects. (**D**) The use of different bone graft substitutes inside the caps. Images (**A**–**C**) Reprinted from Ref. [22], and illustration (**D**) Reprinted from Ref. [23].

This optimized rat model was then adapted and widely applied in our subsequent studies with various modifications. Various studies have employed different cap dimensions, with diameters ranging from 4.4 to 5.0 mm and heights ranging from 1.5 to 3.0 mm (Figure 1A). In the initial setup, plastic caps were placed directly over critical-size defects. In subsequent studies, the caps were fitted into circular grooves containing five perforations, promoting marrow communication (Figure 1B). In some studies, the lids of the caps were either removed or replaced with alternative barrier materials (Figure 1C), while other studies explored the use of different bone graft substitutes inside the caps (Figure 1D). These refinements expanded the model’s applicability and precision in evaluating bone regeneration strategies under diverse conditions.

## 3. Bone Substitute

A series of studies using our rat calvarial GBR model demonstrated the osteogenic potential of different bone-substitute materials, which were evaluated using both micro-CT and histology (Table 1). The aforementioned studies of Kochi et al. indicated that filling plastic caps with HA granules significantly increased bone volume compared to the empty control, as measured via micro-CT [21]. In their comparative analysis, micro-CT and histomorphometry revealed highly consistent patterns of bone regeneration, with mineralized tissue almost reaching the top of the cap in the HA-filled sites but reaching only approximately half the height in the controls after 12 weeks. Quantitative analysis demonstrated a strong correlation between micro-CT and histology results, validating micro-CT as a reliable method for assessing bone regeneration in this model.

Oginuma et al. (2012) compared autogenous bone (AB) to a composite of AB with HA granules [24]. Micro-CT analysis revealed that the bone volume increased in both AB and composite groups, exhibiting no significant differences. Surprisingly, histological evaluation at 12 weeks revealed that the percentage of mineralized tissue was significantly higher in the AB-only group (27.6% ± 7.3%) than in the AB + HA group (45.5% ± 10.4%, *p* < 0.05). These findings suggest that while HA is biocompatible and osteoconductive, the addition of HA may reduce the extent of mineralized bone formation compared with AB alone.

Ozawa et al. (2018) compared a hydroxyapatite/collagen composite (HA/Col) scaffold to an absorbable collagen sponge [25]. Micro-CT revealed significantly higher bone volume (BV) and bone mineral density (BMD) in the HA/Col group than in the absorbable collagen sponge group. Histologically, HA/Col promoted the formation of dense, sponge-like trabeculae, while the absorbable collagen sponge exhibited sparse bone islands with substantial voids, indicating the advantage of the reinforced composite scaffold in supporting bone formation.

Senoo et al. (2022) compared a xenograft material and an all-plastic material, using deproteinized bovine bone mineral (DBBM) and carbonate apatite (CO_3_Ap) [26]. While the DBBM and CO_3_Ap groups exhibited comparable bone volumes in micro-CT evaluation (DBBM: 34.0 ± 3.6%; CO_3_Ap: 35.7 ± 3.2%), the CO_3_Ap group demonstrated a significantly higher BMD than the DBBM group (1.92 ± 0.08 g/cm^3^ vs. 1.85 ± 0.10 g/cm^3^, *p* < 0.05). Histological findings revealed that while DBBM particles were often separated by fibrous tissue, CO_3_Ap induced more uniform bone trabeculae with marrow-like spaces, suggesting superior remodeling and integration. Expanding upon these findings, Watanabe et al. conducted a long-term 24-week study using our rat calvarial GBR model and confirmed that CO_3_Ap maintained its integration without any evidence of resorption or inflammatory response [27]. Importantly, histomorphometric analysis revealed that the CO_3_Ap group, particularly when combined with a resorbable PLACL membrane, achieved significantly greater total tissue height and non-calcified tissue thickness than the other groups, indicating enhanced space maintenance and tissue compatibility.

Collectively, these studies successfully assessed the effectiveness of conventional bone-substitute materials in regulating bone regeneration using our rat calvarial GBR model. While autogenous bone remains the gold standard for osteogenesis, advanced substitutes such as HAp/Col composites and CO_3_Ap offer promising performance with distinct advantages in structural organization and mineralization.

Beyond our own findings, recent investigations by other groups have expanded on the observations by introducing advanced scaffold systems, such as smart scaffolds incorporating bioactive glass [28] or shape-memory polymers [29], scaffolds with antimicrobial [30] or anti-inflammatory effects [31], and 3D-printed constructs [32]. Deng et al. developed a digital light-processing 3D-printed bioactive glass scaffold for alveolar ridge reconstruction in Beagle dogs [32]. This customized bioactive glass scaffold promoted uniform bone formation and stable integration with host tissue, illustrating the potential of additive manufacturing combined with bioactive ceramics to achieve personalized, load-bearing graft substitutes. These emerging scaffold technologies warrant further validation through our standardized rat calvarial GBR model to assess their osteogenic potential under controlled experimental conditions.

## 4. Growth Factor and Hormonal Modulator

The application of growth factors and hormonal modulators has emerged as a promising strategy to enhance bone regeneration in GBR [33]. Using our rat calvarial GBR model, several studies have evaluated the efficacy of these bioactive agents, such as platelet-derived growth factor (PDGF), parathyroid hormone (PTH), and recombinant human basic fibroblast growth factor-2 (rhFGF-2), in promoting vertical bone augmentation beyond the skeletal envelope (Table 2).

Tsuchiya et al. (2013) investigated the effects of PDGF-BB delivered at concentrations of 0.01% and 0.03% via an absorbable collagen sponge [34]. Both doses significantly increased bone volume and vertical bone height compared to the control group, as assessed via micro-CT and histological analysis. By 12 weeks, the radiopacity in the PDGF-treated groups reached the full height of the plastic cap, whereas in the control group, it was confined to one-third of the cap. Histomorphometric measurements confirmed significantly larger bone area (71.8% and 62.4% for 0.01% and 0.03% PDGF, respectively) and vertical bone height (95.3% and 90.9%, respectively) compared to controls (34.7% and 48.4%). These findings underscore that angio- and vasculogenic cells may function as important targets that are the initial responders to the application of this mitogenic factor, facilitating bone growth within the confined GBR space.

Most recently, Kogure et al. (2025) reported that 0.3% rhFGF-2 incorporated into an atelocollagen sponge significantly enhanced vertical bone augmentation in the same rat calvarial model [23]. Starting from week 8, micro-CT analyses showed a significant increase in bone volume compared to controls. At 12 weeks, histomorphometry revealed that the area and height of new bone formation for the rhFGF-2 group were 35.6% and 41.9%, whereas those for the control group were only 9.1% and 13.4%, respectively. The newly formed bone exhibited a trabecular architecture at both the periosteal and dural surfaces. These results confirm the dual role of rhFGF-2 in promoting angiogenesis and osteoblast precursor proliferation, even in the absence of mineralized bone grafts.

While PDGF-BB and rhFGF-2 were examined as local bioactive agents to accelerate bone regeneration, Tsunori et al. examined the intermittent systemic administration of PTH at two dosages (35 µg/kg and 105 µg/kg, three times per week) over a 12-week period [35]. In contrast to the localized approaches, PTH was administered intraperitoneally, and no bone-substitute material was used. Micro-CT imaging revealed a dose-dependent increase in bone volume: radiopacity extended to one-third, one-half, and two-thirds of the plastic cap height in the control, PTH-35, and PTH-105 groups, respectively. Histological sections showed thicker lamellar bone and increased numbers of Runx2-positive osteoblasts in the PTH groups. The high-dose group exhibited significantly greater new bone formation, suggesting a robust anabolic effect of systemic PTH that can enhance bone formation even without scaffold materials.

Together, these studies highlight the potential of combining space-maintaining scaffolds with potent biological agents to achieve vertical bone regeneration. While PDGF-BB and rhFGF-2 act locally via scaffold-mediated delivery, primarily modulating cellular proliferation and angiogenesis, intermittent PTH functions systemically to stimulate osteoblast activity and bone remodeling. Each of these agents significantly enhances bone regeneration within the GBR space, validating their utility in preclinical models. These findings provide compelling biological evidence for the clinical translation of growth factor-based GBR strategies for predictable vertical bone augmentation.

Although BMPs have not yet been examined in our rat GBR model, special attention is warranted among growth factors, as recombinant human BMP-2 (rhBMP-2) has already obtained regulatory approval for alveolar ridge and GBR applications [36]. In our previous rabbit study wherein a titanium-cap model was employed, rhBMP-2 marked vertical bone augmentation beyond the skeletal envelope in a clear dose-dependent manner [14]. Bone formation reached approximately two-thirds of the cap height, whereas only limited bone formation was observed in the control group. In contrast, Thoma et al. compared rhBMP-2 and rhPDGF-BB in a rabbit calvarial model using polycarbonate cylinders filled with DBBM. After eight weeks, the rhBMP-2 group achieved nearly complete space filling and more than double the mineralized bone fraction compared with the rhPDGF-BB group, confirming the superior osteoinductive potential of rhBMP-2 and the suitability of DBBM as its carrier [37]. Among these agents, BMP-2 stands out as a clinically validated osteoinductive molecule that bridges preclinical discovery and clinical application, underscoring the importance of assessing rhBMP-2 within our standardized rat calvarial model.

## 5. Mechanical Barriers

Conventionally, the concept of GBR has emphasized the use of rigid occlusive barriers to prevent soft-tissue invasion and create an isolated compartment for osteogenic cells. However, recent evidence suggests that rather than promoting new bone formation, complete occlusion may restrict the beneficial infiltration of blood supply, growth factors, and progenitor cells necessary for robust bone regeneration. Several experimental studies using rat calvarial models have clarified how barrier permeability critically modulates the balance between soft-tissue exclusion and biological communication with the surrounding environment (Table 3).

Yamamoto et al. (2018) systematically investigated the influence of barrier permeability by comparing occlusive plastic caps, open caps, and caps with three or four holes [22]. Micro-CT and histological analyses demonstrated that bone augmentation was inversely associated with barrier permeability: the open group exhibited minimal bone formation, while occlusive and three-hole caps supported significantly greater bone volume. Importantly, Masson’s trichrome staining revealed that the quality of the regenerated bone—measured via collagen maturation—was highest in the three-hole group. This indicated that a moderately permeable barrier may offer the most favorable conditions, preventing excessive soft-tissue invasion while permitting ingress of biological cues from the periosteum and marrow.

Extending this concept, Senoo et al. (2022) compared microporous and macroporous titanium meshes in a similar rat GBR model [26]. Microporous meshes (20-μm pore diameter) promoted significantly more new bone formation (approximately 38–40% of the region of interest (ROI)) than macroporous meshes (31.5%). Histological observations confirmed that microporosity facilitated angiogenesis and microvasculature formation while limiting soft-tissue invasion. In contrast, macroporous meshes with millimeter-scale pores allowed fibrous tissue ingrowth, which compromised osteogenesis. These findings suggest that pore size in the micrometer range helps achieve an optimal balance between vascular infiltration and osteogenic stability, redefining the role of mechanical barriers as semipermeable rather than strictly occlusive.

Most recently, Watanabe et al. (2024) examined the performance of a newly developed resorbable bilayer PLACL (poly-L-lactic acid/ε-caprolactone) membrane in a rat GBR model [27]. Compared with conventional collagen membranes, PLACL maintained structural integrity and barrier function for up to 24 weeks, with partial degradation observed only at later stages. In particular, the combination of CO_3_Ap and PLACL significantly increased the total tissue height and non-calcified tissue compared with DBBM plus collagen. The total tissue height and non-calcified tissue height were significantly higher in the PLACL group than in the collagen group, whereas no significant difference in calcified tissue height was observed between the groups. These findings indicate that durable, resorbable membranes with controlled permeability can support both space maintenance and biological communication during prolonged healing, overcoming the limitations of earlier resorbable membranes that degraded too rapidly. Therefore, the PLACL membrane is expected to not only promote bone augmentation through long-term space maintenance but also enhance soft-tissue formation.

Collectively, these studies demonstrate that the success of GBR depends not on maximizing occlusivity but on achieving controlled permeability that allows selective passage of angiogenic and osteogenic factors while preventing fibrous invasion. Clinically, a network meta-analysis of 27 trials comparing 11 membrane types revealed that titanium-reinforced d-PTFE achieved the greatest vertical bone gain but carried higher risks of exposure and soft-tissue injury, whereas resorbable collagen membranes exhibited fewer complications [38]. These data highlight that mechanical stability alone is insufficient for predictable regeneration—tissue integration and permeability are equally significant.

A recent innovation is the biodegradable magnesium membrane, which combines mechanical strength with complete resorbability. Elad et al. introduced the magnesium-membrane shield technique for immediate dentoalveolar bone regeneration [39]. The magnesium membrane maintains space during healing, gradually converts to magnesium salts, and releases hydrogen gas that temporarily supports soft tissue, preventing collapse. Clinically, it enables reconstruction of compromised buccal or palatal walls with immediate implant placement, resulting in stable cortical bone and uneventful soft-tissue healing. Owing to its strength, malleability, and bioresorbability, magnesium offers a promising alternative to titanium meshes and polymer membranes for simultaneous space maintenance and biological integration [40,41]. This emerging membrane must be validated in our standardized rat calvarial GBR model to confirm its osteogenic potential under controlled conditions.

## 6. Risk Factors and Systemic Conditions

The increasing demand for implant-based treatments, coupled with the demographic shift toward an aging population, has highlighted the need for a stronger biological evidence base to address the influence of systemic conditions on the success of vertical GBR (Table 4). Saito et al. investigated the impact of nicotine administration in our rat calvarial GBR model, in which plastic caps were placed to create an augmented space [42]. Rats that received systemic nicotine injections at doses comparable to those received by typical human smokers exhibited significantly reduced bone volume and height gains compared with saline-treated controls. Micro-CT revealed delayed and diminished radiopacity within the augmentation space in nicotine-exposed animals, while histological sections showed thinner lamellar bone and fewer osteoblast-like cells and microvessels. Although bone augmentation beyond the skeletal envelope was not eliminated completely, the overall regenerative outcome was substantially compromised. These findings indicate that nicotine jeopardizes but does not entirely prevent bone formation, largely through suppression of osteogenesis, inhibition of angiogenesis, and reduction of key osteogenic and angiogenic mediators.

Kubota et al. (2018) examined the role of estrogen deficiency using our GBR model in ovariectomized rats [43]. In vivo micro-CT analysis demonstrated that compared with controls, newly formed bone volume within the augmentation space was significantly reduced in estrogen-deficient animals. Histological analysis revealed sparse and thinning trabeculae, abundant non-calcified spaces, and reduced connectivity. Immunohistology showed that RUNX2-positive osteoblast-like cells were scattered, and COL-I-positive fibers were weakly expressed, indicating impaired osteoblast differentiation and collagen matrix formation. These results demonstrate that estrogen deficiency directly hampers osteoblast differentiation and extracellular-matrix production, thereby compromising bone augmentation.

Building on these results, Kubota et al. (2018) evaluated whether intermittent systemic administration of PTH can be used to enable bone regeneration in ovariectomized rats [44]. Guided bone augmentation was performed with plastic caps, and rats were treated with intermittent intraperitoneal injections of PTH (40 μg/kg, three times per week). Micro-CT analysis revealed that the bone volume in the PTH-treated OVX group was significantly higher than that in the untreated OVX controls and even exceeded that of healthy sham-operated animals. Histological sections exhibited newly formed bone occupying nearly 50% of the augmentation space in PTH-treated OVX rats, compared to 12% in untreated OVX rats and 23.6% in healthy controls. Runx2-positive osteoblast-like cells were abundant not only adjacent to existing bone but also within regenerated tissue remote from parent bone, while TRAP-positive osteoclasts were modestly increased. These findings indicate that intermittent PTH exerts a robust anabolic effect, stimulating osteoblast proliferation and differentiation, and can overcome the impaired bone regeneration associated with estrogen deficiency.

Current evidence derived from our model is restricted to nicotine exposure and estrogen deficiency. Further investigations addressing other systemic and patient-related factors, including diabetes, metabolic syndrome, chronic inflammation, and immunosenescence, are required to comprehensively delineate their influence on GBR outcomes. Other research groups have investigated the impact of diabetes on GBR with preclinical calvaria studies. Retzepi et al. investigated the effects of experimental diabetes and glycemic control on GBR using a rat calvarial critical-size defect model [45]. In this defect-based GBR model, standardized 5 mm parietal defects were covered with ePTFE membranes on both intracranial and extracranial sides to create a secluded healing environment. Despite severe hyperglycemia, considerable bone regeneration occurred even in rats with uncontrolled diabetes; however, quantitative analysis revealed markedly delayed defect closure and reduced new bone area compared with healthy controls at 15 and 30 d. Gene expression profiling showed significant downregulation of osteogenic and angiogenic genes (*BMP4*, *LTBP4*, *THRA*, *CD276*) in uncontrolled diabetic cases. These findings suggest that uncontrolled diabetes affects early phases of bone regeneration following GBR. Lee et al. evaluated the effects of experimental diabetes and metabolic control under titanium domes with hydrophobic or hydrophilic surfaces using their rat calvarial model [46]. While substantial bone formation was observed even under uncontrolled diabetic conditions, the difference in dome materials and surface characteristics critically influenced osteogenesis. Because the biological response and regenerative dynamics depend significantly on the material properties of the space-maintaining device, the findings obtained from titanium-dome models cannot be directly extrapolated to our resin cap GBR system. Future investigations using our standardized model will be essential to clarify how diabetes and other systemic factors modulate vertical bone regeneration.

## 7. Biological Potential of Regenerated Bone

The question of whether GBR-regenerated bone possesses regenerative functions comparable to those of the native cortical bone remains an unresolved question with direct clinical relevance, especially when considering the reuse of augmented tissues formed during osteoplasty or implant-site preparation. Addressing this issue, Kubota et al. (2017) established a two-stage protocol in rats in which augmented bone was first generated under a plastic cap over the right calvarium for 12 weeks, and then, in secondary surgery, particulate grafts harvested from this augmented bone were transplanted into a critical-size defect on the contralateral side [47]. The outcomes were compared to those of defects grafted with cortical-bone particulates and to those of ungrafted controls [47]. Longitudinal micro-CT indicated that for both defects grafted with augmented-bone particulates and those with cortical-bone particulates, radiopacity remained consistently high from 0 to 12 weeks, whereas controls exhibited only a gradual, limited increase. Quantitatively, bone volume within the ROI for grafted groups was maintained at approximately 9–11 mm^3^ at all times and consistently exceeded the bone volume of the controls. Histologically, both-grafted groups exhibited robust reossification, with a preference for dural-side bone formation; however, the cortical-bone particle group achieved superior defect closure to the augmented-bone group (approximately 91% vs. 86% at 12 weeks), while the control group remained largely unhealed. Immunohistochemistry revealed a biological distinction: augmented-bone grafts contained more Runx2-positive osteoblast-like cells and more TRAP-positive osteoclasts than cortical-bone grafts at 12 weeks, indicating an actively remodeling yet relatively immature state of the regenerated tissue. Considered together, these findings confirm that while bone generated within a GBR space is biologically competent and can serve as a regenerative autograft source, its regenerative performance, at least for the examined time period, remains inferior to that of cortical bone. This likely reflects an imbalance among osteogenesis, osteoinduction, and osteoconduction in the augmented tissue and the limitations of micro-CT in discriminating particulate remnants from the de novo bone.

Although comparing the biological potential of regenerated bone in this model with that from other GBR models appears reasonable, such comparisons should be made cautiously, as outcomes are influenced by species, age, bone-marrow penetration, and materials used. The present data define the intrinsic regenerative capacity of GBR-derived bone under standardized conditions. Clinically, Cucchi et al. found that regenerated bone in human mandibles following GBR contained approximately 40% new bone and exhibited less trabecular maturity than native bone [48]. These human findings parallel our rat data, indicating that GBR-derived bone, although biologically competent, remains histologically immature compared with cortical bone even after several months of healing.

## 8. Future Perspectives

Conventionally, GBR has relied on rigid barriers to exclude soft tissue and establish a compartment for bone regeneration [49]. However, the specific cellular activities occurring within this regenerative space have not been thoroughly investigated as cellular or genetic events. In this context, the integration of our GBR model and newly developed single-cell spatial omics approach provides an unprecedented opportunity for in situ visualization of the cellular and molecular events that occur within GBR sites [50,51]. These approaches elucidate how different membranes and bone substitutes affect the regenerative microenvironment, identify the anatomical origins of infiltrating cells, such as the periosteum, dura mater, or marrow compartments, and reveal patterns of cell-to-cell communication, capturing how immune, vascular, and stromal cells interact to orchestrate osteogenesis within the confined GBR space.

Importantly, for mineralized tissues, a recent study on mouse long-bone repair demonstrated that the major limitation for such repair, i.e., the degradation of RNA during decalcification, can be overcome [52]. It was shown that an optimized protocol combining Morse’s-solution decalcification with Visium/CytAssist significantly improves transcript capture and generates high-quality spatial maps across fracture healing stages, resolving periosteal progenitors, their transcriptional regulators, and ligand–receptor interactions guiding macrophage recruitment and osteochondral differentiation. These advances indicate that spatial transcriptomics, supported by appropriate sample preparation, can robustly capture the dynamics of bone regeneration [53].

From a practical perspective, adopting spatial omics in our GBR program also raises the question of species choice. While our rat model remains highly informative for biomechanical assessment, volumetric quantification, and histology, the current single-cell and spatial ecosystem is considerably more advanced for mice because most vendor panels, probe sets, and antibody libraries are mouse-oriented, and mice offer extensive genetic tools for lineage tracing and functional perturbation. Thus, the establishment of a mouse GBR model with comparable geometry can facilitate access to these resources while maintaining the ability to interrogate regenerative events under standardized conditions.

Advances in material science—including 3D and 4D printing [54,55,56], stimuli-responsive materials [57,58], and ion-releasing bioceramics [59]—further expand the potential of GBR systems to interact dynamically with their biological environment. Incorporating these innovations will enable precise control of space architecture, degradation kinetics, and biofactor release, allowing direct testing of how mechanical and biochemical cues influence the cellular programs identified by means of spatial omics.

Translational research remains essential for linking preclinical findings with clinical practice. Our standardized rat calvarial GBR model provides a reproducible and ethically feasible platform for systematic evaluation of biomaterials and biological modulators, helping explain the clinical behavior of materials such as collagen membranes and carbonate apatite. Looking ahead, integrating this framework with next-generation tools such as digital pathology, single-cell sequencing, and spatial transcriptomics will deepen our understanding of the host response to regenerative implants and accelerate the translation of biomaterials toward safer and more predictable clinical outcomes [60].

## 9. Conclusions

Fifteen years of rat calvarial GBR research has taught us that true bone regeneration does not depend on filling space but rather requires the orchestration of different biological functions. Our model captures how materials, molecules, and systemic factors shape bone healing. The next step is to advance from measuring bone volume to decoding the cellular symphony. We believe that spatial omics is ideally suitable for studies of cellular signals and will play a key role in guiding the future development of predictable, biology-informed regeneration.

## Figures and Tables

**Table 1:** Overview of research on bone substitutes.

Study (Author, Year)	Bone Substitute(s)	Micro-CT Findings	Histological Findings	Key Conclusion
Kochi et al., 2009 [20]	HA vs. Empty	The HA group showed significant time-dependent bone volume increase; new bone reached the cap top. Control had bone at ~half height new bone formed beyond the skeletal envelope. In contrast, there was no bone formation beyond the skeletal envelope at the control site.	HA bridged defect edges; no vertical augmentation in empty control.	HA performed as a scaffold for vertical bone augmentation beyond the skeletal envelope.
Kochi et al., 2010 [21]	HA vs. Empty	Micro-CT and histology showed consistent regeneration; strong correlation.	The HA group had more mineralized tissue than control. At 12 weeks, histology showed that the HA group had significantly higher percentages and height of newly generated and mineralized tissue than the control.	Micro-CT is reliable for longitudinal bone regeneration analysis.
Oginuma et al., 2012 [24]	AB vs. AB + HA	Both increased bone volume similarly.	AB-only group had significantly higher mineralized tissue (27.6%) than AB+HA (45.5%, *p* < 0.05).	HA may interfere with early mineralization when mixed with AB.
Ozawa et al., 2018 [25]	HA/Collagen Composite vs. Collagen sponge	The HA/Collagen Composite group reached approximately twice compared with collagen sponge.	HA/Collagen induced dense trabeculae; collagen sponge showed sparse bone with voids.	The HA/Collagen composite is superior to collagen sponge for bone augmentation.
Senoo et al., 2022 [26]	DBBM vs. CO_3_Ap	Similar bone volume (DBBM: 34.0%, CO_3_Ap: 35.7%), but BMD was higher in CO_3_Ap (1.92 g/cm^3^ vs. 1.85 g/cm^3^, *p* < 0.05).	CO_3_Ap: uniform trabecular bone; DBBM: fibrous encapsulation between particles.	CO_3_Ap supports better bone integration and remodeling.
Watanabe et al., 2024 [27]	DBBM or CO_3_Ap + PLACL or Collagen Membrane	CO_3_Ap + PLACL showed favorable height and space maintenance at 24 weeks.	CO_3_Ap + PLACL induced thick non-calcified tissue, no inflammation.	CO_3_Ap effective long-term; PLACL membrane ensures prolonged barrier function.

HA: hydroxyapatite. AB: autogenous bone. DBBM: deproteinized bovine bone mineral. CO_3_Ap: carbonate apatite. PLACL: poly(L-lactic acid) and poly(ε-caprolactone).

**Table 2:** Overview of research on growth factors and hormonal modulators.

Study (Author, Year)	Bioactive Agent	Delivery Method (Scaffold Included)	Micro-CT Findings	Histological Findings	Key Conclusion
Tsuchiya et al. (2013) [34]	PDGF-BB (0.01%, 0.03%)	Local (with collagen sponge)	Increased BV; plastic cap full at 4 w (0.03%) and at 12 w (0.01%); 1/3 of the plastic cap full at 12 w (control)	BV: 71.8% (0.01%), 62.4% (0.03%) 34.7% (control); height: 95.3% (0.01%), 90.9% (0.03%), 48.4% (control)	PDGF-BB promotes bone formation beyond the skeletal envelope.
Kogure et al. (2025) [23]	rhFGF-2 (0.3%)	Local (with collagen sponge)	Increased BV; a rapid increase started at 8 w; by 12 w, approximately half of the ROIs were within the caps	Increased BV; 0.3% rhFGF-2: 35.6% (area) and 41.9% height at 12 w; control: 9.1% (area) and 13.4% height at 12 w	With proper space maintenance, rhFGF-2 can effectively promote vertical bone regeneration without requiring additional bone mineral particles.
Tsunori et al. (2023) [35]	PTH (35, 105 µg/kg)	Systemic (intraperitoneal injection without scaffold)	Dose-dependent increase: ~1/3 (control), ~1/2 (PTH-35), ~2/3 (PTH-105)	Thicker lamellar bone; more Runx2+ osteoblasts in PTH groups	Intermittent PTH systemically enhances osteogenesis in a dose-dependent manner without a scaffold.

**Table 3:** Overview of mechanical-barrier research.

Study (Author, Year)	Barrier Type	Micro-CT Findings	Histological Findings	Key Conclusion
Yamamoto et al., 2018 [22]	Plastic caps: Occlusive/Open/3-hole/4-hole	Bone volume inversely correlated with permeability. Occlusive > 3-hole > 4-hole > Open.	The 3-hole group exhibited the highest level of collagen maturation (Masson’s trichrome).	Moderate permeability (e.g., 3-hole) optimizes balance between soft tissue exclusion and biological signaling.
Senoo et al., 2022 [26]	Titanium mesh: Microporous (20 μm) vs. Macroporous (1–2 mm)	Microporous: ~38–40% new bone; Macroporous: ~31.5%.	Microporous: Less fibrous ingrowth, rich microvasculature Macroporous: Fibrous ingrowth	Microporous barriers provide optimal vascular entry while preventing fibrous invasion.
Watanabe et al., 2024 [27]	Resorbable bilayer membrane: PLACL vs. Collagen	Bone volume increased over time in all groups, with no significant differences among them.	Total tissue height and non-calcified tissue height: Significantly higher in the PLACL group than in the Collagen group.	The PLACL membrane is expected to not only promote bone augmentation through long-term space maintenance but also enhance soft-tissue formation.

**Table 4:** Overview of research on the effects of risk factors and systemic conditions.

Study (Author, Year)	Systemic Factor	Experimental Group	Micro-CT Findings	Histological Findings	Key Conclusion
Saito et al., 2012 [42]	Nicotine	Nicotine injection (systemic)	Significantly reduced bone volume and height gains, with delayed and diminished radiopacity in the augmented space	Thinner lamellar bone, fewer osteoblast-like cells, fewer microvessels	Nicotine compromises but does not abolish bone formation via inhibition of osteogenesis and angiogenesis.
Kubota et al., 2018 [43]	Estrogen Deficiency (OVX)	OVX rats	Significantly reduced bone volume in augmented space compared to controls	Sparse, thinning trabeculae; abundant non-calcified areas; reduced RUNX2+ and COL-I+ osteoblast activity	Estrogen deficiency impairs osteoblast differentiation and ECM production, compromising bone augmentation.
Kubota et al., 2018 [44]	Estrogen Deficiency + Rescue Therapy (PTH)	OVX rats + intermittent PTH	Bone volume significantly higher than untreated OVX and healthy controls	50% bone fill (OVX + PTH) vs. 12% (untreated OVX) and 23.6% (sham); abundant RUNX2+ cells; mild increase in TRAP cells	Intermittent PTH restores bone regeneration in OVX rats, indicating strong anabolic osteogenic effects.

## Data Availability

No new data were created or analyzed in this study. Data sharing is not applicable to this article.

## Abbreviations

The following abbreviations are used in this manuscript:

GBR	Guided Bone Regeneration
HA	Hydroxyapatite
HA/Col	Hydroxyapatite/Collagen Composite
CO_3_Ap	Carbonate Apatite
AB	Autogenous Bone
DBBM	Deproteinized Bovine Bone Minera
BMD	Bone Mineral Density
BV/TV	Bone Volume/Total Volume
PDGF(-BB)	Platelet-Derived Growth Factor (-BB subtype)
rhFGF-2	Recombinant Human Basic Fibroblast Growth Factor-2
PTH	Parathyroid Hormone
TRAP	Tartrate-Resistant Acid Phosphatase
RUNX2	Runt-Related Transcription Factor 2
COL-I	Collagen Type I
PLACL	Poly-L-lactic acid/ε-caprolactone
OVX	Ovariectomized
micro-CT	Micro-Computed Tomography
ROI	Region of Interest
